# Comparison of urethral sling surgery and non-ablative vaginal Erbium:YAG laser treatment in 327 patients with stress urinary incontinence: a case-matching analysis

**DOI:** 10.1007/s10103-021-03317-x

**Published:** 2021-04-22

**Authors:** Nobuo Okui, Hironari Miyazaki, Wataru Takahashi, Toshihide Miyauchi, Chikako Ito, Machiko Okui, Kaori Shigemori, Yoshiharu Miyazaki, Zdenko Vizintin, Matjaž Lukac

**Affiliations:** 1 Urology, Kanagawa Dental University, Yokosuka, Kanagawa Japan; 2 Urology, Dr Okui’s Urogynecology and Urology, Yokosuka, Kanagawa Japan; 3 Urology, Teikyo University, Tokyo, Tokyo Japan; 4 Urology, Koshigawa Hospital, Dokkyo University, Saitama, Saitama Japan; 5 Urology, Yokosuka City Hospital, Yokosuka, Kanagawa Japan; 6 Urology, Yakuin Urogenital Clinic, Fukuoka, Fukuoka Japan; 7 Urology, Kengun Kumamoto Urology, Kumamoto, Kumamoto Japan; 8 Urology, Kumamoto University, Kumamoto, Kumamoto Japan; 9 Urology, Ooita Urology Hospital, Ooita, Ooita Japan; 10 Urology and Gynecology, Saint Sofia Clinic, Nagoya, Aichi Japan; 11 Fotona d.o.o., Stegne 7, 1000 Ljubljana, EU Slovenia

**Keywords:** Propensity score analysis, Stress urinary incontinence, Tension-free vaginal tape, Non-ablative vaginal Erbium:YAG laser treatment

## Abstract

Stress urinary incontinence (SUI) occurs when abdominal pressure, such as from coughing or sneezing, causes urine leakage. We retrospectively compared tension-free vaginal tape (TVT) and non-ablative vaginal Erbium:YAG laser treatment (VEL) by propensity score (PS) analysis in women with SUI. No PS analysis studies have investigated urethral sling surgery using polypropylene TVT and VEL for SUI. Data from patients aged 35–50 years who were treated for SUI and registered at several institutions were selected. Patients with medical records covering 1 year for the 1-h pad test, who completed the International Consultation on Incontinence Questionnaire-Short Form (ICIQ-SF) and the Overactive Bladder Symptom Score (OABSS), were included. We analyzed 102, 113, and 112 patients in the TVT, VEL, and control groups, respectively. Compared with the control group, the TVT and VEL groups exhibited significant improvement in the 1-h pad test and ICIQ-SF. In the PS analysis, the TVT and VEL groups similarly improved in the 1-h pad test and ICIQ-SF. As for the OABSS, the VEL group showed significantly greater improvement than the TVT group. In the odds ratio analysis for the 1-h pad test, no differences in any of the parameters were observed between TVT and VEL. VEL may be considered an alternative to TVT for SUI treatment.

**Supplementary Information:**

The online version contains supplementary material available at 10.1007/s10103-021-03317-x.

## Introduction

Stress urinary incontinence (SUI) occurs when abdominal pressure such as that from coughing or sneezing causes urine leakage [1]. It affects up to 49% of all women. Mixed urinary incontinence (MUI) affects up to 29% of all women [2] and is a combination of SUI and urge incontinence, in which a strong urge to urinate is accompanied by urinary leakage.

SUI is commonly treated with mid-urethral sling surgery using polypropylene mesh tape. Tension-free vaginal tape (TVT) surgery is a typical procedure for SUI. These procedures have shown good treatment outcomes. However, there is a long-term risk of complications due to the artificial nature of the tape [3].

Recently, patient concerns over the implantation of artificial objects [3] have made it necessary to develop new therapies for incontinence. This has led to research on methods for vaginal and urethral irradiation with lasers, including non-ablative vaginal Erbium:YAG laser treatment (VEL) [4]. VEL uses an Erbium:YAG laser, which has a 2940-nm wavelength absorbed by water. By adjusting the laser’s pulse mode, the mucosa can be uniformly heated without heat accumulation on the surface layer [5, 6], raising temperatures to 45 °C deep in the tissue and 67 °C on the surface. The treatment promotes cellular synthesis by improving collagen remodeling and blood flow, and it has been reported to improve urinary incontinence [7–9].

While a comparison of TVT and VEL would be meaningful, thus far, the literature only includes one prospective study evaluating incontinence only, which was conducted by our research group [10]. In the present study, we retrospectively extracted TVT and VEL cases from the Japanese medical records to establish a database containing all SUI procedures and outcomes. We have named this database the Urinary Incontinence Research Network (UnIcoRN) database, and it was used throughout this study to retrospectively analyze SUI propensity score (PS) [11].

Recent International Consensus Group recommendations suggest that laser therapy should be restricted to research until further robust data are available [12–14]. On July 30, 2018, the United States Food and Drug Administration (FDA) issued a warning against the use of energy-based devices (EBD), including laser, to perform “vaginal rejuvenation” or vaginal cosmetic procedures. However, 2 years and many new studies later, a systematic review was published in the *Menopause* journal [15] in which the authors stated that “the FDA safety communication appears unsubstantiated” adding that “The well-documented benefits and low risk of adverse events suggest laser therapy for GSM is reasonable with appropriate pretreatment counseling.”

The therapeutic advantages of nonsurgical laser-based devices in urogynecology can only be recommended after robust clinical trials have demonstrated their long-term complication profile, safety, and efficacy. Recently, a global survey about the safety of non-ablative vaginal Erbium laser was published [16], reporting about the safety of this technology used over a period of more than 8 years on more than 113,000 patients in 535 clinics from 43 countries, showing an excellent safety profile of this laser. In the future, we need more robust clinical trials with protocols based on data analysis that has already been carried out. By accurately analyzing already conducted clinical data, this study will be useful for future controlled trials. Moreover, given the international concerns about the artifacts used in the middle sling procedure, it is ethically impossible for vaginal lasers and middle sling procedures to continue in randomized controlled trials. For this reason, PS comparison with past data is very meaningful.

## Materials and methods

### Study design

The present study compared the effects of TVT and VEL on urinary incontinence using retrospective analysis of the UnIcoRN database, which is a comprehensive database in Japan that we have established to compare VEL to other treatments. Medical records of all registered cases were examined. The TVT group was compared to the VEL group using PS, and the advantages of TVT and VEL were evaluated using odds ratio meta-analysis.

### Patients

SUI was diagnosed based on the International Consultation on Incontinence definition as a “Complaint of involuntary loss of urine on effort or physical exertion (e.g., sporting activities), or on sneezing or coughing” [17] by urological specialists. All identified cases were compared to a control group.

The subjects were patients between 35 and 50 years of age at the time of treatment who (1) underwent TVT surgery, (2) received VEL treatment, or (3) were placed under observation with no treatment (control) for SUI at several facilities, within a period of 15 years between 2004 and 2019. Patients with medical records covering a period of at least 1 year after treatment were registered. The choice of treatment type (VEL or TVT) was up to the patients after the consultations, at which they were informed in detail about both options.

The cost of the laser procedure for this study was the same as the middle sling procedure. For this reason, the price was not a factor on which the patients would choose between the two treatments.

The following selection criteria were applied to the registered patients: (1) availability of medical records before and at 1 year after treatment for the 1-h pad test [18]; (2) completion of the International Consultation on Incontinence Questionnaire-Short Form (ICIQ-SF) [19]; (3) completion of the Overactive Bladder Symptom Score (OABSS) [20]; (4) availability of records from before treatment (including complete information on age; body mass index [BMI] [21–23]; marital status; number of deliveries; desire for children; history of medications for hypertension [23–25], diabetes [24, 25], cerebral infarction [24, 25], and hyperlipidemia [24]); and (5) availability of histories of cerebral infarction, smoking [24, 26], spinal disease, pelvic surgery [26], and menopause [24, 26]. The exclusion criteria were as follows: (1) hospitalization for diseases other than SUI during the 1-year observation period; (2) ongoing female hormone replacement therapy; (3) presence of cystocele, uterine prolapse, or rectocele [20]; and (4) use of overactive bladder (OAB) medications or anticholinergic agents. All patients performed pelvic floor muscle training [PFMT] daily [17].

The UnIcoRN study was launched to document all VEL treatments performed for all types of urinary incontinence (SUI, MUI, and urinary urgency incontinence [UUI]). Sample size was calculated using the Raosoft tool (Raosoft Inc., WA, USA). The sample size for the three groups was estimated to provide a 95% confidence interval with a 5% margin of error.

We examined 112, 159, and 159 Asian patients who underwent the same treatment protocol for TVT+PFMT, VEL+PFMT, and PFMT alone, respectively. Of these, 102, 113, and 112 patients without missing data and aged between 35 and 50 years were included in the TVT, VEL, and control groups, respectively. The drop-out rate, i.e., patients who were excluded because of the data missing in their charts, was calculated using Fisher’s *F*-test. The *F*-test showed that the differences between TVT vs VEL, VEL vs control, and TVT vs control were 0.27, 0.99, and 0.27, respectively. There were no statistically significant differences between the three groups.

### Treatments and analysis

TVT surgery [10] was performed by specialists under lumbar anesthesia or general anesthesia using the Advantage Fit™ Transvaginal Mid-Urethral Sling System (Boston Scientific Co., MA, USA) or the GYNECARE TVT™ Retropubic System (Ethicon Inc., NJ, USA). The study was conducted for 1 year, with the day of operation being designated as day 0.

VEL treatment [10] was performed using a standard protocol at all institutions. A non-ablative Erbium:YAG laser (FotonaSmooth™ XS; Fotona d.o.o., Ljubljana, Slovenia) was used. The vagina was sprayed with 9% xylocaine, after which a glass speculum made for the laser was inserted into the vagina; the wavelength was set to 2940 nm and irradiation was performed for 20 min in a special long-pulse mode. Irradiation was performed first on the entire anterior vaginal wall at 6 J/cm^2^ for 10 min, then on the entire vagina at 3.0 J/cm^2^ for 5 min, and around the urethra at 10 J/cm^2^ for 5 min. Laser irradiation was performed every other month for three treatment sessions [10]. The study was conducted for 1 year, with the day of the first VEL treatment being designated as day 0.

The surgical procedures for both types of sling as well as the VEL settings and treatment procedures were the same throughout the study period in all centers included in this study.

The daily PFMT [17] was supervised by urology specialists for all patients in the three groups. In the control group, the study was conducted for 1 year, with the day of the first lesson being designated as day 0.

The primary endpoint was ≤1 g in the 1-h pad test at 1 year after treatment. This was regarded as a cure for urinary incontinence [3]. The secondary endpoint was improvement in the total ICIQ-SF and OABSS.

Comparisons between the three groups (TVT, VEL, and control) were performed using the Kruskal-Wallis test [11] after the Kolmogorov-Smirnov test. Comparisons between the two groups (TVT, VEL) were performed using the Mann-Whitney *U* test [11]. Hospital visitation records of the three groups were checked for the number of patients and the results of the 1-h pad test, ICIQ-SF, and OABSS at 0, 3, 6, 9, and 12 months after the first day of treatment. Comparisons between 0 and 12 months were performed using the Mann-Whitney *U* test [11]. A *p* value of <0.05 was considered to indicate a significant difference.

For PS estimation [11], we used a logistic regression model in which the treatment status (TVT or VEL) was regressed on the following baseline characteristics: age; BMI; marital status; number of deliveries; menopause status; desire for children; medications for hypertension, diabetes, cerebral infarction, or hyperlipidemia; smoking habits; and a history of spinal disease, breast cancer, or pelvic surgery. BMI ≥25 kg/m^2^ was considered obese.

Some studies have shown that PS methods result in biased estimates of conditional odds ratios for binary outcomes [27]. We analyzed this study data with multivariate analysis, which can be found in the supplement’s figures and tables.

Improvement to ≤1 g in the 1-h pad test at 1 year after treatment was used in the Peto odds ratio meta-analysis.

The statistical software R version 2.15.1 (R Core Team, Vienna, Austria) and Microsoft Excel version 1911 (Microsoft Corp., WA, USA) on a Windows 10 version 1903 (Microsoft Corp.) operating system were used for all analyses.

## Results

### Patient characteristics

Table 1 summarizes the characteristics of patients in the TVT and VEL groups and focuses on the major differences between patients who chose TVT or VEL in actual clinical practice. A significant difference was only observed for “desire for children” (11.8% for TVT vs 50.4% for VEL, *p* < 0.001) and “number of deliveries” (1.3 for TVT vs 1.1 for VEL, *p* = 0.015). Between the two treatment groups, women who wanted children selected VEL significantly more often than TVT (*p* < 0.001). The medical records of patients who chose VEL were examined to determine the reasons for this; in all cases, their reason was that they “wanted VEL or to just wait and not have surgery, out of concern the artificial implant from TVT surgery would be a problem in pregnancy and delivery.”

**Table 1:** Demographics and populations of the three treatment groups

Parameter	TVT group (*n* = 102)	VEL group (*n* = 113)	Control group (*n* = 112)	*p* value*	*p* value**
				(Total)	(TVT vs VEL)
Age (years)	42.5 (35–48)	42.7 (37–49)	43.3 (38–48)	0.275	0.417
Body mass index (kg/m^2^)	23.2 (19–25.5)	22.9 (20–25.6)	22.8 (20–25.6)	0.366	0.178
Married (partner)	76.5%	73.5%	75.0%	0.878	0.612
No. of deliveries	1.3 (0–4)	1.1 (0–4)	1.3 (0–4)	0.015	0.015
Menopause	11.8%	11.5%	8.9%	0.754	0.954
Desire for children	11.8%	50.4%	42.9%	<0.001	<0.001
Hypertension	0.9%	1.8%	1.8%	0.863	0.627
Diabetes	2.0%	1.8%	3.6%	0.635	0.922
Cerebral infarction	1.0%	1.8%	1.8%	0.863	0.627
Hyperlipidemia	2.0%	1.8%	3.6%	0.863	0.627
Smoking	13.7%	13.3%	13.4%	0.995	0.925
Spinal disease	0%	0%	0%	1.0	1.0
Breast cancer	0%	0%	0%	1.0	1.0
Pelvic surgery	2.0% 2 ovarian cysts	2.7% 1 ovarian cyst, 1 uterine cancer, 1 uterine fibroid	1.9% 1 ovarian cyst, 1 uterine cancer	0.894	0.74
1-h pad test	31.6 g (15–60 g)	29.9 g (14–60 g)	34.3 g (12–62 g)	0.128	0.054
ICIQ-SF	12.1 (8–21)	11.2 (7–21)	12.0 (8–21)	0.0924	0.0612
OABSS	1.83 (0–10)	2.24 (0–11)	1.7 (0–10)	0.892	0.821

Mean, minimum, and maximum values are shown for age, body mass index, number of deliveries, 1-h pad test, ICIQ-SF, and OABSS. Percentages of patients taking medication for hypertension, diabetes, cerebral infarction, and hyperlipidemia are shown. Percentages of patients with a history of pelvic surgery and the names of the main diseases (number of patients) are shown

*The Kruskal-Wallis test was used to compare the three groups (TVT, VEL, and control groups)

**The Mann-Whitney *U* test was used to compare the two groups (TVT and VEL groups)

### Treatment courses

**Fig. 1:**
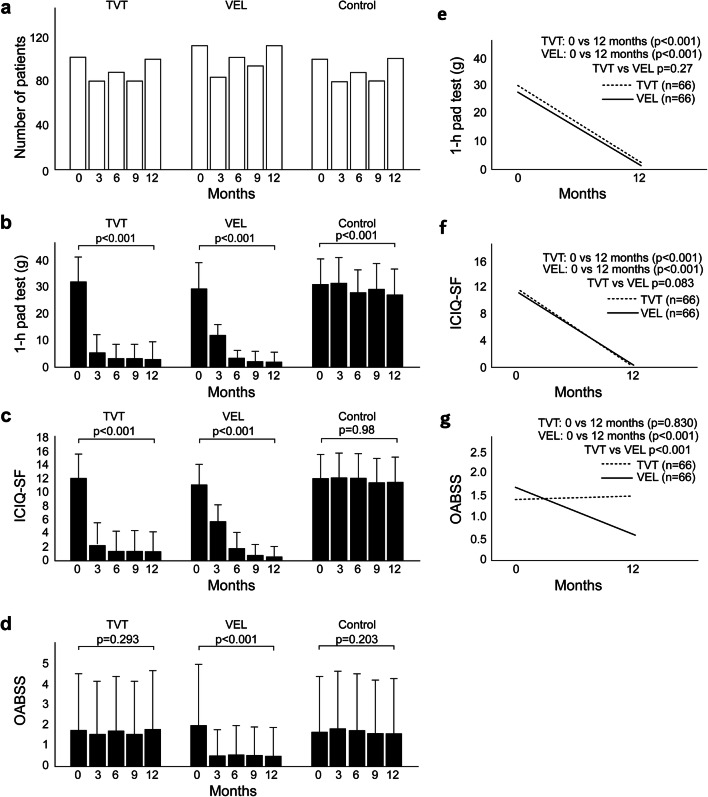
Treatment in the TVT and VEL groups. **a** The number of patients at 0 months, 3 months, 6 months, 9 months, and 12 months in three groups. **b**–**d** The change over time for the 1-h pad test, ICIQ-SF, and OABSS. There was a significant difference between the start of treatment and 1-year post-treatment in the TVT and VEL groups. No significant difference was observed in the control. **e**–**g** Comparison between TVT and VEL with respect to changes in outcomes from pretreatment (0 months) to post-treatment (1 year). Only OABSS was significantly different between the two groups. The Mann-Whitney *U* test was used to compare the three groups at 1 and 12 months post-treatment

We focused on the treatment courses of all registered cases. Figure 1a shows the number of patients, and Fig. 1b and c present the results of the 1-h pad test and ICIQ-SF, respectively, both of which significantly improved from 0 to 12 months in the TVT and VEL groups (*p* < 0.001). In the supplement (Fig. 3), we also show the box plot comparison of the TVT group (*n* = 102) and VEL group (*n* = 113) for the 1-h pad test at 1 year after treatment. Figure 1d shows the changes in OABSS. Overactive bladder (OAB) was observed in 39 out of 102 patients in the TVT group and 41 out of 113 patients in the VEL group. While OABSS declined to 0 in 19 patients in the VEL group, this did not occur for any patient in the TVT group, which had two new OAB cases. In the supplement (Fig. 4), we show the change in Q3 and Q4 from “before” to “1 year.” There was no significant difference in the TVT group (Q3: *p* = 0.16, Q4: *p* = 0.50). In the VEL group, Q3 and Q4 improved significantly (Q3 < 0.05, Q4 < 0.05).

In this study, 34 patients were treated with the Advantage Fit, and 68 patients were treated with the GYNECARE TVT™ sling. The operation times were 58.0 ± 10.2 vs 60.0 ± 7.3 min (*p* = 0.28). Bleeding times were 38.2 ± 9.0 vs 36.4 ± 8.7 min (*p* = 0.32). These data also show that we provided a uniform procedure for all mid-urethral sling patients.

### Outcomes

We compared the TVT and VEL groups with respect to changes in outcomes from pretreatment (0 months) to post-treatment (1 year). Sixty-six pairs were matched using PS. Figure 1e and f present the results of the 1-h pad test and ICIQ-SF. Both groups exhibited improvements from 0 to 12 months, although the difference between the two therapies was not significant. This indicates that in terms of these two tests, TVT and VEL are equivalent. However, OABSS was significantly different between the VEL and TVT groups (*p* < 0.001; Fig. 1g). OAB was observed in 20 out of 66 patients in the TVT group and 20 out of 66 patients in the VEL group. At 12 months, OAB was noted in 22 out of 66 patients in the TVT group and 13 out of 66 patients in the VEL group. Two patients in the TVT group had both de novo urgency and de novo UUI.

### Advantages

**Fig. 2:**
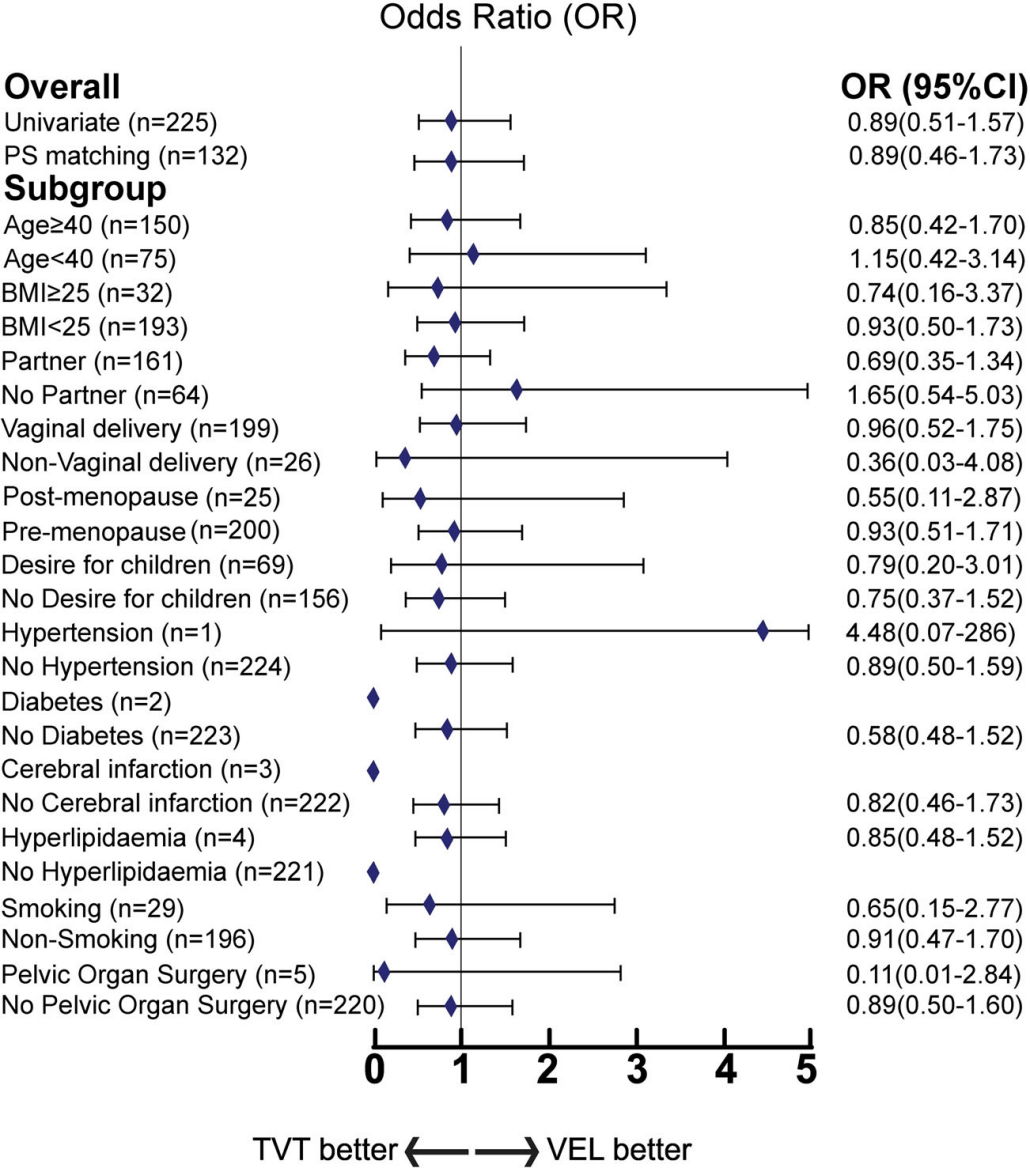
Advantages of TVT and VEL in Peto odds ratio meta-analysis. Comparison of 1-h pad test from TVT and VEL. Comparisons were performed between the registered samples, and the samples were statistically processed by PS and the PS subgroups. Patients were divided according to 1-h pad test results, with ≤1 g considered cured, and higher values not cured. TVT was considered superior if the odds ratio was <1.0. VEL was considered superior if the odds ratio was larger than 1.0. TVT, tension-free vaginal tape. VEL non-ablative vaginal Erbium:YAG laser treatment, PS propensity score, CI confidence interval

We focused on the advantages of TVT and VEL. Figure 2 divides patients according to whether incontinence was cured or not, with ≤1 g in the 1-h pad test regarded as cured. TVT and VEL had almost the same effect on incontinence. However, the small sample sizes for hypertension, diabetes, cerebral infarction, and hyperlipidemia made it impossible to compare TVT and VEL.

We show the multivariate analysis in the supplement (Tables 2 and 3).

## Discussion

### Main findings

All VEL treatments performed in Japan between 2004 and 2019 were investigated. We compared TVT and VEL as treatments for SUI. In this retrospective study, both methods performed better than controls. Using PS matching, TVT and VEL outcomes were equivalent with respect to the 1-h pad test and ICIQ-SF, while VEL was better than TVT with respect to OABSS. The subgroup analysis showed that TVT did not have a statistically significant advantage in most situations. VEL was particularly effective in patients who did not have a sexual partner. These were important confirmations, as there have been no previous comparative studies with PS retrospectively analyzed in SUI.

### Interpretation

Based on our current and previous prospective study [10], the VEL and TVT procedures are comparable for SUI; however, laser therapy is superior in terms of MUI. Even after PS matching, de novo UUI and de novo urgency appeared after treatment in the TVT group. With regard to OAB, including UUI and urgency, we saw improvement in the VEL group. In our previous study, we found that some cases of severe MUI worsened with TVT but improved with VEL [10]. The incidence of de novo urgency at 1 year after TVT was reported to be 22.2% [28]. On the other hand, VEL was not reported to generate de novo urgency or any serious major adverse events [29].

Notably, VEL is inferior to TVT with respect to the time that it took to obtain a cure. A desired effect was observed immediately after surgery with TVT, while this took several months with VEL. This highlights the distinction between using an artificial object in TVT, while VEL involves the proliferation of living cells [30, 31].

To the best of our knowledge, the physiological mechanisms underlying VEL treatment are still unknown [28, 30–32]. The results of our current study indicate that the mechanism of action in VEL differs from that of TVT [32]. We have previously shown that the mechanism of VEL differs from that of OAB medications and possibly involves improved blood flow in vaginal and bladder tissues [31]. Several studies have shown that pelvic ischemia and oxidative stress may play a main role in lower urinary tract dysfunction (LUTD), including detrusor overactivity (DO)/overactive bladder (OAB) and detrusor underactivity (DU)/underactive bladder (UAB) [33, 34]. At the 2019 International Consultation on Incontinence-Research Society (ICI-RS) meeting [34], this topic was extensively discussed and compared with ischemia of other organs, and the development of pelvic ischemia in animal models [35]. Collagen remodeling from VEL is associated with the induction of revascularization and improved blood flow in tissues [30, 31, 36]. This offers a new field of treatment for managing SUI.

Recently, the important role of blood flow in the urethra and bladder has been noted [37]. Stem cells have been used in treatment of stress urinary incontinence [38]; in a study using adipose-derived regenerative cells, a cytokine release assay showed that adherent cells secreted cytokines associated with angiogenesis, including vascular endothelial growth factor-A, angiopoietin-2, and placental growth factor. Using a stem cell therapeutic approach [39] for pelvic floor disorders (PFD), studies using different autologous stem cells have achieved promising results by improving the pelvic ligament and muscle regeneration and conferring tissue elasticity and strength to the damaged tissue in PFD, as well as reducing inflammatory reactions, collagen deposition, and foreign body reaction [39]. However, these studies were not enough to fully evaluate function and side effects. Better quality studies are needed to document the exact mechanism of action, longevity, safety, and its eventual place in the current treatment protocols of SUI and OAB. Artificial urinary sphincter (AUS) in female SUI has been reported for decades, but due to its challenging implantation and inherent morbidity, in most countries it was not in use [40]. Over the past few years, laparoscopic and, more recently, robotic techniques of AUS implantation in female patients have been described with promising perioperative outcomes. The use of AUS in female patients has been restricted to certain countries and a few high-volume centers. PFMT is being studied in combination with electromyography biofeedback (providing visual or auditory feedback of internal muscle movement) [41]. Female patients were positive about both interventions; adherence to the interventions was facilitated by a desire to improve their urinary incontinence and hindered by lack of time. However, one report showed that at 24 months, no evidence was found of any important difference in the severity of urinary incontinence between the PFMT plus electromyographic biofeedback and PFMT only groups [42]. Routine use of electromyographic biofeedback with PFMT should not be recommended. Other ways of maximizing the effects of PFMT should be investigated [42].

Finally, the uncertainty in modern society that surrounds surgical insertion of artificial objects is an important factor [3]. In the present study, we demonstrated how the motivation of patients who had a desire to have children in the future affected their choice of treatment. We focused on the changes in patients who wanted a sexual partner. VEL could represent an alternative option for such patients who are concerned about the artificial implants used in TVT.

### Limitations

This report included a sample population limited to a single ethnicity. There is no published article containing a comparison study of race/ethnicity for incontinence treatment. With pelvic surgical procedures, African American, Hispanic, and Asian/Pacific Islander women eligible for minimally invasive hysterectomy were more likely than white women to receive abdominal hysterectomy [43]. One review shows that extraordinary risk factors such as ethnicity and race, mixed and fecal incontinence, and iatrogenic and neurogenic factors should be discussed in a follow-up report [44]. The results may also be influenced by Asian physical and psychological characteristics.

The physiological mechanism of VEL treatment could not be demonstrated using this study’s methodology. In future studies, we need long-term data and more cases [28, 32].

## Conclusion

This study demonstrates that both TVT and VEL are viable options for patients desiring SUI treatment. VEL may be an option for patients with both SUI and OAB symptoms, as TVT can worsen urinary urgency and frequency, and VEL could represent an option for patients who are concerned about artificial implants.

## Supplementary Information

ESM 1 Box plot comparison between the TVT group (n= 102) and VEL group (n=113) for the 1-hour pad test at 1 year after treatment. In the Figure, these plots contain sample size, medians, ranges with outliers, and the 25th and 75th percentiles. (JPG 57 kb)

ESM 2 OABSS-Q3 (Urinary Urgency), Q4 (Urinary Urgency Incontinence; UUI) at before treatment and 1 year after treatment. In the TVT group, the F-test before vs 1 year. The squares are 1 to 5 points in Q3 or Q4, represented by: no urge (point 1), mild (point 1, 2), moderate (point 3, 4) and severe (point 5). In this study, there were no severe MUI (point 5) patients. (JPG 95 kb)

ESM 3 (DOCX 46 kb)

ESM 4 (DOCX 45 kb)
