# Supplementary figures and images for: Comparison of urethral sling surgery and non-ablative vaginal Erbium:YAG laser treatment in 327 patients with stress urinary incontinence: a case-matching analysis
Source: Lasers Med Sci. 2021 Apr 22;37(1):655–63. doi: 10.1007/s10103-021-03317-x (PMC8803680; doi:10.1007/s10103-021-03317-x)

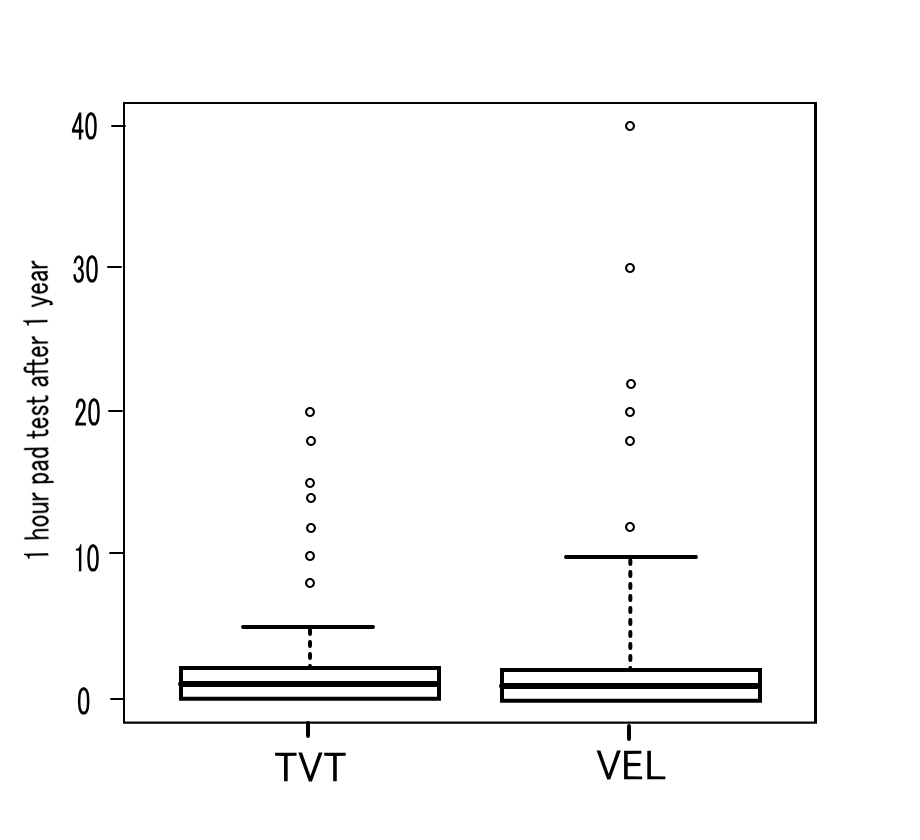

Supplement: Supplementary file 1 — Box plot comparison between the TVT group (n= 102) and VEL group (n=113) for the 1-hour pad test at 1 year after treatment. In the Figure, these plots contain sample size, medians, ranges with outliers, and the 25th and 75th percentiles. (JPG 57 kb) [file 10103_2021_3317_MOESM1_ESM.jpg]

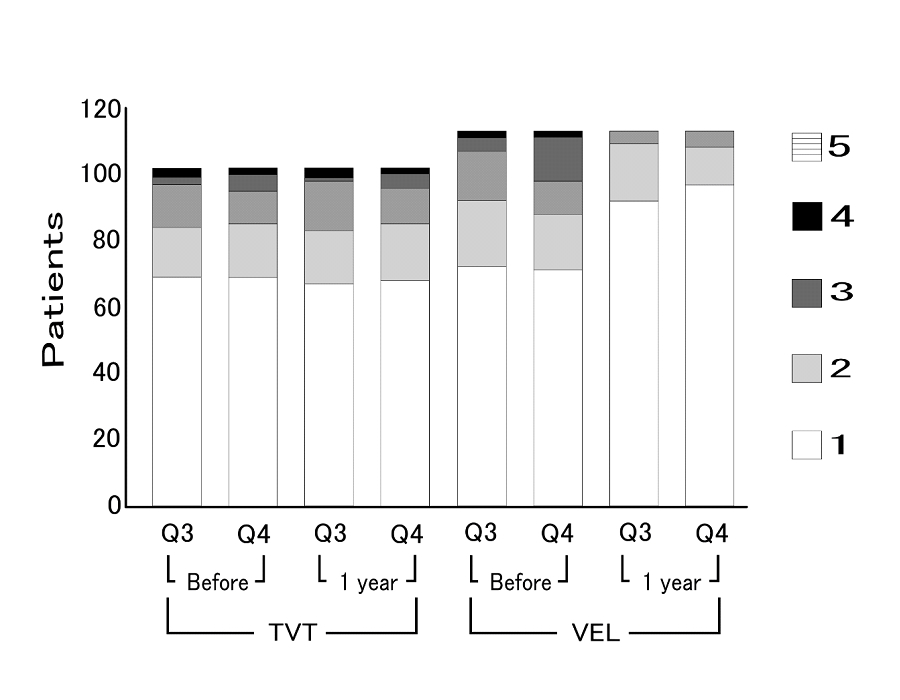

Supplement: Supplementary file 2 — OABSS-Q3 (Urinary Urgency), Q4 (Urinary Urgency Incontinence; UUI) at before treatment and 1 year after treatment. In the TVT group, the F-test before vs 1 year. The squares are 1 to 5 points in Q3 or Q4, represented by: no urge (point 1), mild (point 1, 2), moderate (point 3, 4) and severe (point 5). In this study, there were no severe MUI (point 5) patients. (JPG 95 kb) [file 10103_2021_3317_MOESM2_ESM.jpg]
