# Supplementary material for: Comparison of urethral sling surgery and non-ablative vaginal Erbium:YAG laser treatment in 327 patients with stress urinary incontinence: a case-matching analysis
Source: Lasers Med Sci. 2021 Apr 22;37(1):655–63. doi: 10.1007/s10103-021-03317-x (PMC8803680; doi:10.1007/s10103-021-03317-x)
Supplement: Supplementary file 3 — (DOCX 46 kb) [file 10103_2021_3317_MOESM3_ESM.docx]

**Table 1.** Demographics and populations of the three treatment groups.

| Parameter | TVT group (n=102) | VEL group (n=113) | Control group (n=112) | p-value* | p-value** |
| --- | --- | --- | --- | --- | --- |
|  |  |  |  | (Total) | (TVT vs. VEL) |
| Age (years) | 42.5 (35-48) | 42.7 (37-49) | 43.3 (38-48) | 0.275 | 0.417 |
| Body mass index (kg/m^2^) | 23.2 (19-25.5) | 22.9 (20-25.6) | 22.8 (20-25.6) | 0.366 | 0.178 |
| Married (Partner) | 76.5% | 73.5% | 75.0% | 0.878 | 0.612 |
| No. of deliveries | 1.3 (0-4) | 1.1 (0-4) | 1.3 (0-4) | 0.015 | 0.015 |
| Menopause | 11.8% | 11.5% | 8.9% | 0.754 | 0.954 |
| Desire for children | 11.8% | 50.4% | 42.9% | <0.001 | <0.001 |
| Hypertension | 0.9% | 1.8% | 1.8% | 0.863 | 0.627 |
| Diabetes | 2.0% | 1.8% | 3.6% | 0.635 | 0.922 |
| Cerebral infarction | 1.0% | 1.8% | 1.8% | 0.863 | 0.627 |
| Hyperlipidemia | 2.0% | 1.8% | 3.6% | 0.863 | 0.627 |
| Smoking | 13.7% | 13.3% | 13.4% | 0.995 | 0.925 |
| Spinal disease | 0% | 0% | 0% | 1.0 | 1.0 |
| Breast cancer | 0% | 0% | 0% | 1.0 | 1.0 |
| Pelvic surgery | 2.0%  2 ovarian cysts | 2.7%  1 ovarian cyst  1 uterine cancer  1 uterine fibroid | 1.9%  1 ovarian cyst  1 uterine cancer | 0.894 | 0.74 |
| 1-h pad test | 31.6 g (15 g-60 g) | 29.9 g (14 g-60 g) | 34.3 g (12 g-62 g) | 0.128 | 0.054 |
| ICIQ-SF | 12.1 (8-21) | 11.2 (7-21) | 12.0 (8-21) | 0.0924 | 0.0612 |
| OABSS | 1.83 (0-10) | 2.24 (0-11) | 1.7 (0-10) | 0.892 | 0.821 |

Mean, minimum, and maximum values are shown for age, body mass index, number of deliveries, 1-h pad test, ICIQ-SF, and OABSS. Percentages of patients taking medication for hypertension, diabetes, cerebral infarction, and hyperlipidemia are shown. Percentages of patients with a history of pelvic surgery and the names of the main diseases (number of patients) are shown.

*The Kruskal-Wallis test was used to compare the three groups (TVT, VEL, and control groups)

**The Mann-Whitney U test was used to compare the two groups (TVT and VEL groups)
