# Supplementary material for: Comparison of urethral sling surgery and non-ablative vaginal Erbium:YAG laser treatment in 327 patients with stress urinary incontinence: a case-matching analysis
Source: Lasers Med Sci. 2021 Apr 22;37(1):655–63. doi: 10.1007/s10103-021-03317-x (PMC8803680; doi:10.1007/s10103-021-03317-x)
Supplement: Supplementary file 4 — (DOCX 45 kb) [file 10103_2021_3317_MOESM4_ESM.docx]

**Table 2. (**supplement table)

|  | Odds ratio | Lower 95% CI | Upper 95% CI | p-value |
| --- | --- | --- | --- | --- |
| Age | 1.04 | 0.956 | 1.14 | 0.350 |
| Intervention  TVT:1  VEL:0 | 0.84 | 0.468 | 1.52 | 0.573 |
| Hypertension  Yes:1  No:0 | 4.19e-07 | 0.000 |  | 0.990 |
| Hyperlipidemia  Yes:1  No:0 | 3.35e+13 | 0.000 |  | 0.984 |
| Menopause  Yes:1  No:0 | 1.06 | 0.381 | 2.93 | 0.918 |
| Child delivery  Yes:1  No:0 | 0.99 | 0.288 | 3.37 | 0.982 |
| Pelvic floor surgery  Yes:1  No:0 | 1.32 | 0.21 | 8.32 | 0.765 |

Logistic regression analysis of comparison between the TVT group (n= 102) and VEL group (n=113). The objective variable is the 1-hour pad test (≤1 g :0, others :1) at the 1 year after treatment. The explanatory variables are intervention (TVT or VEL), hypertension, menopause, child delivery, and pelvic floor surgery.
